# Emergence of a novel sequence type carbapenem-resistant hypervirulent *Klebsiella pneumoniae* ST6417 harboring *bla*_NDM-5_ on the lncX3 plasmid

**DOI:** 10.1128/spectrum.00984-24

**Published:** 2024-08-20

**Authors:** Junnian Liu, Sue Yuan, Luhan Xuan, Yu Sun, Xinyu Zhang, Lu Qiao, Xuefei Du

**Affiliations:** 1 Clinical Laboratory, The Fourth Affiliated Hospital of Harbin Medical University, Harbin, Heilongjiang, China; Universidad de Buenos Aires, Buenos Aires, Argentina

**Keywords:** *Klebsiella pneumoniae*, ST6417, carbapenem resistance gene, hypervirulent, IncX3

## Abstract

Carbapenem-resistant hypervirulent *Klebsiella pneumoniae* (CR-hvKP) is a significant pathogen causing major public health problems worldwide. This study characterized a novel sequence type 6417 (ST6471) CR-hvKP strain recovered from the blood of a male patient with septicemia. Strain CR2021 is not susceptible to carbapenems, cephalosporin, sulfonamides, quinolones, or levofloxacin and is susceptible to amikacin and tigecycline. Molecular typing indicated that ST6417 is derived from the most dominant hypervirulent *K. pneumoniae* (hvKP) clone in China, ST23, with a single-locus variation in *tonB*. The genomic characterization of CR2021, which contains three plasmids, was performed through whole-genome sequencing. The plasmid pCR2021_IncFII contains 12 antibiotic resistance genes [*bla*_CTX-M-3_, *bla*_TEM-1B_, *bla*_DHA-1_, *aac (3)-Ild*, *aadA16*, *sul1*, *sul2*, *qnrB4*, *ARR-3*, *dfrA27*, *qacE*, *merACDE*], all of which are associated with genetic elements. The plasmid pCR2021_IncFIB carries crucial virulence-related genes, while the plasmid pCR2021_IncX3 only harbors the *bla*_NDM-5_ resistance gene and exhibits 99% similarity with two other *bla*_NDM-5_-carrying IncX3 plasmids (pSHX180-NDM5, pNDM-K725), with coverage of 87% and 100%, respectively. The *bla*_NDM-5_ genetic region contains an additional IS26-Tn3 genetic module. Serum killing and anti-human neutrophil phagocytosis tests indicated that CR2021 exhibits high virulence, which was further confirmed in a *Galleria mellonella* larvae infection model. CR-hvKP is becoming more prevalent in China; however, the majority have evolved from the multidrug resistance clone ST11 and its variants by acquiring virulence factors. Conversely, CR-hvKP derived from hvKP, such as the clone ST23, remains relatively rare. Therefore, the discovery of ST6417 underscores the need for further research into the genetic characteristics and evolution of bacteria.

**IMPORTANCE:**

ST11 and its variants, which often exhibit drug resistance, represent popular clones of carbapenem-resistant hypervirulent *Klebsiella pneumoniae* (CR-hvKP) in China, often leading to high morbidity and mortality rates owing to their high virulence and robust drug resistance. Conversely, CR-hvKP, originating from the high-virulence sequence type ST23, remains rarely reported. In this study, we identified a novel ST6417 CR-hvKP strain derived from ST23, carrying *bla*_NDM-5_ on an IncX3 plasmid conferring resistance to carbapenems. In addition, we elucidate its virulence, resistance to drugs, and genetic characteristics. The discovery of ST6417 highlights the diverse pathways in the evolution of CR-hvKP, warranting increased attention.

## INTRODUCTION

Hypervirulent *Klebsiella pneumoniae* (hvKP) is a more aggressive pathogen compared to classical *K. pneumoniae* (cKP), often causing community infections in healthy individuals, leading to various severe diseases, including pneumonia, endophthalmitis, meningitis, and pyogenic liver abscess (1). In previous studies, hvKP was considered susceptible to clinically employed antibiotics; however, due to the widespread dissemination of drug-resistant mobile elements, hvKP has gradually developed resistance to most antibiotics. Carbapenem-resistant hypervirulent *K. pneumoniae* (CR-hvKP) has emerged even clinically (2, 3). Among the various mechanisms giving rise to CR-hvKP, the production of carbapenemases is the most significant (4). Studies focusing on genes encoding carbapenemases have elucidated *bla*_KPC_ as the most common, followed by *bla*_NDM_. Carbapenase genes are frequently flanked by many transposons and insertion elements, facilitating their transfer between plasmids, and thereby promoting the spread of multidrug-resistant (MDR) strains (5, 6).

Among CR-hvKP strains widely prevalent in China, sequence type 11 (ST11) is the predominant clone and has been confirmed to be significantly correlated with multidrug resistance (7). Unlike ST11, ST23 is considered to be closely related to hvKP in China. An increasing number of CR-hvKP strains of diverse sequence types based on these two sequence types have emerged, showing more complex virulence and drug resistance characteristics. Therefore, further investigations into CR-hvKP-related data are urgently needed to better prevent and treat infections caused by CR-hvKP (8, 9).

In this study, we elucidate the genetic, virulence, and drug resistance characteristics of a clinical CR-hvKP strain harboring the *bla*_NDM-5_ drug-resistance gene, classified as a novel sequence type, ST6417, arising from a C-to-A mutation at locus 239 of the *tonB* housekeeping gene of ST23.

## RESULTS

### Clinical information and an *in vitro* drug-sensitivity test

The patient carrying strain CR2021 was a 78-year-old male admitted to the hospital on 1 August 2020, because of coronary atherosclerotic heart disease and myocardial infarction. One week after admission, his body temperature continued to rise to 38°C, leading to a diagnosis of pneumonia and cholecystitis. Despite clinical treatment for diseases caused by various bacterial infections with aztreonam, meropenem, imipenem, tigecycline, gentamicin sulfate, and cefoperazone/sulbactam sodium, the patient’s condition progressed to bacteremia. Strain CR2021 was isolated from a blood sample. Unfortunately, the patient eventually succumbed to septic shock with multiple organ failure on 7 November 2020. Isolation and purification of strain CR2021 followed immediately through *in vitro* drug-sensitivity tests, which showed that CR2021 was not sensitive to piperacillin/tazobactam, cefepime, cefoxitin, ceftazidime, ceftriaxone, ertapenem, imipenem, sulfamethoxazole, levofloxacin, amoxicillin/clavulanic acid, gentamicin, aztreonam, meropenem, and other antibiotics, except for amikacin and tigecycline (Table 1).

**TABLE 1:** *In vitro* drug sensitivity tests of strain CR2021

Antibacterial agent	MIC	Rad	Interpretation^*a*^
Tigecycline	1		S
Cefuroxime sodium	≥64		R
Piperacillin/tazobactam	≥128		R
Cefepime	≥32		R
Cefuroxime axetil	≥64		R
Cefoxitin	≥64		R
Cefoperazone/sulbactam	≥64		R
Ceftazidime	≥64		R
Ceftriaxone	≥64		R
Ertapenem	≥8		R
Imipenem	≥16		R
Amikacin	≤2		S
Sulfamethoxazole	≥320		R
Levofloxacin	4		R
Amoxicillin/clavulanic acid	≥32		R
Gentamicin		6	R
Aztreonam		10	R
Ampicillin/sulbactam		6	R
Meropenem		10	R

^*a*^ S, susceptible; R, resistant.

### Multilocus sequence typing and capsular serotyping

Multilocus sequence typing (MLST) and capsular serotyping results showed that the sequence type of CR2021 is ST6417 with capsular serotype K1. The housekeeping gene sequence spectrum included *gapA*, *infB*, *mdh*, *pgi*, *phoE*, *rpoB*, and *tonB* (loci 2, 1, 1, 1, 9, 4, 893) compared with ST23 (loci 2, 1, 1, 1, 9, 4, 12); only the *tonB* changed from 12 to 893. Sanger sequencing confirmed that the 12-893 transition was caused by a C-to-A mutation in the *tonB* 239 locus.

### Genomic characterization of strain CR2021

**Fig 1:**
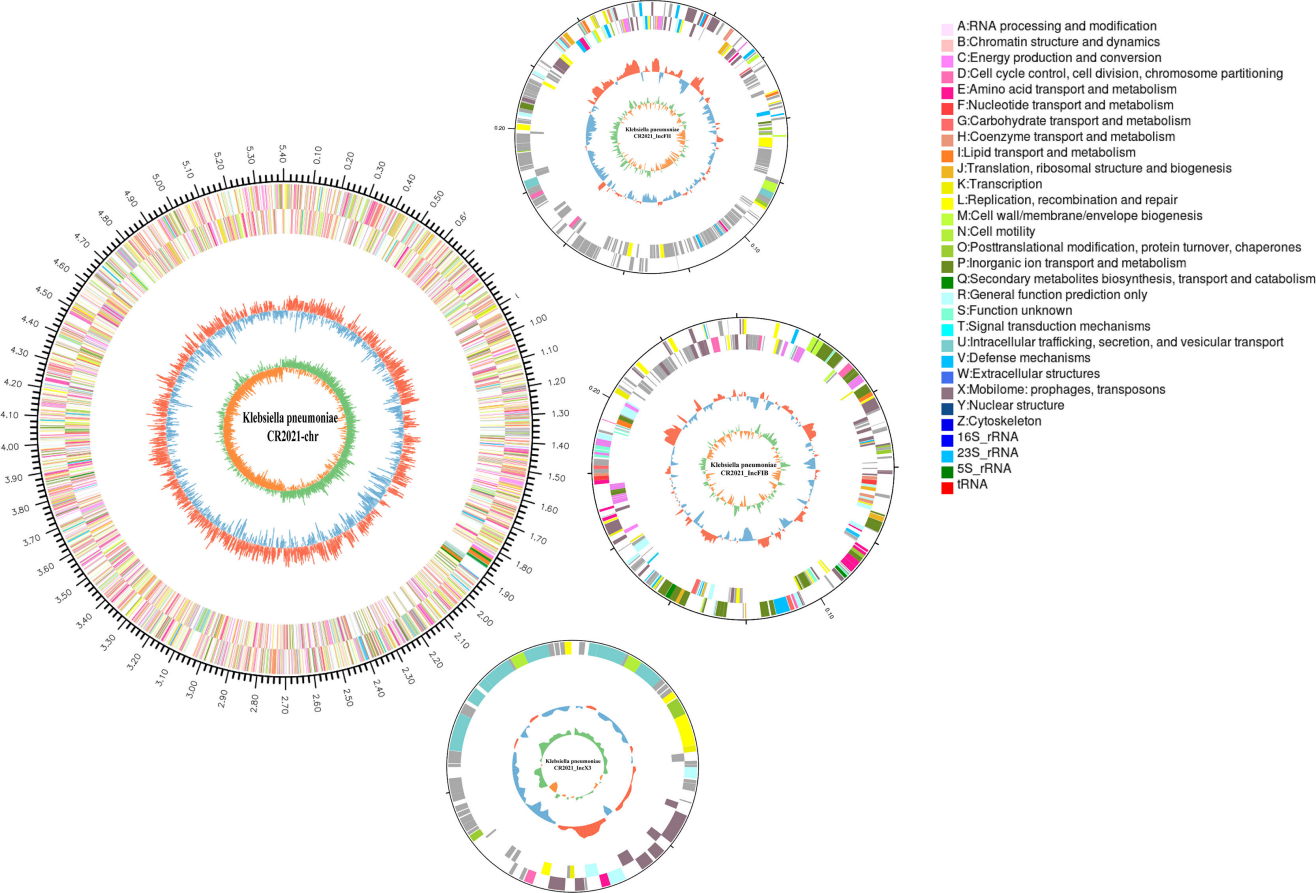
Circular map of the chromosome and plasmids of strain CR2021. The outermost circle represents the genome size. The second and third circles depict CDS on the positive and negative strains, respectively. Different colors indicate the functional classification of different Clusters of Orthologous Groups (COG) of CDS. The fourth circle represents rRNA and tRNA. The fifth circle represents the GC content. The outward red segment indicates regions with higher GC content compared to the average GC content of the entire genome; the higher the peak value, the greater the difference between the GC content and the average GC content. The inward blue segment indicates regions with lower GC content compared to the average GC content of the entire genome; the higher the peak value, the greater the difference between the GC content and the average GC content. The innermost circle represents the GC-skew value.

Detailed information regarding the genome of strain CR2021 was obtained through whole-genome sequencing and bioinformatics analysis. Strain CR2021 comprised a 5.2 Mb chromosome and three plasmids of sizes 263 kb, 242 kb, and 56 kb, respectively, designated as CR2021-chr, pCR2021_IncFII, pCR2021_IncFIB, and pCR2021_IncX3, respectively (Fig. 1). The Guanine and Cytosine (GC) content of CR2021-chr sequences was higher (57.46%), containing 4,946 predicted coding sequences (CDS), 87 tRNAs, and 25 rRNAs. Compared to that of the chromosome, the GC content of the plasmid sequences was lower (pCR2021_IncFII, 46.63%; pCR2021_IncFIB, 50.09%; pCR2021_IncX3, 46.47%), and the predicted CDS were 288, 268, and 70 (Table 2).

**TABLE 2:** Chromosome and plasmid genome characteristics of strain CR2021

Structure name^*a*^	Size (bp)	GC content (%)	CDS	tRNA	rRNA	Resistance gene	Virulence gene
CR2021-chr	5,404,496	57.46	4,946	87	25	*bla*_SHV-190_, *fos*A6, *PhoPQ*, *oqxAB*, *Ompk35*, *Ompk37*	*fimABCDEFGHI*, *mrkABCDFHIJ*, *ybt*, *fyu*, *iroBD*, *entABCDEF*, *allS*
pCR2021_IncFII	263,252	46.63	288			*bla*_CTX-M-3_, *bla*_TEM-1B_, *bla*_DHA-1_, *aac*(*3*)-*Ild*, *aadA16*, *sul1*, *sul2*, *qnrB4*, *ARR-3*, *dfrA27*, *qacE*, *merACDE*	
pCR2021_IncFIB	241,557	50.09	268			*pcoABCDRS*, *SilABCDPRS*, *terABCDZ*	*iroBCDN*, *iucABCD*-*iutA*
pCR2021_IncX3	55,817	46.47	70			*bla*_NDM-5_	

^*a*^ chr: chromosome; IncFII, IncFIB, IncX3: the type of plasmid.

The structural components (*TSSABCEFGHJKLM*, *VarG*, and *PAAR*) of the type VI secretion system (T6SS), along with six genes closely associated with bacterial virulence, including the allantoin gene (*allS*), type I fimbrial-related genes (*fimABCDEFGHI*), type III fimbrial-related genes (*mrkABCDFHIJ*), the siderophore-associated genes *ybt* and *fyu* (encoding yersiniabactin), *iroBD* (encoding salmonellin), and *entABCDEF* (encoding enterobactin), were identified on CR2021-chr. In addition, five genes linked to drug resistance were found on the chromosome: genes for *β*-lactam resistance (*bla*_SHV-190_), fosfomycin resistance (*fosA6*), macrolide, peptide antibiotic resistance (*PhoPQ*), efflux pump protein (*oqxAB*), and membrane porins (*Ompk35* and *Ompk37*).

Prediction of the genes on the plasmid pCR2021_IncFII revealed eight antimicrobial resistance genes: genes for *β*-lactam resistance (*bla*_CTX-M-3_, *bla*_TEM-1B_, *bla*_DHA-1_), aminoglycoside resistance (*aac (3)-Ild* and *aadA16*), sulfanilamide resistance (*sul1* and *sul2*), quinolone resistance (*qnrB4*), rifampicin resistance (*ARR-3*), trimethoprim resistance (*dfrA27*), quaternary amine resistance (*qacE*), and metallic mercury resistance (*merACDE*). In addition to carrying the genes for copper resistance (*pcoABCDRS*), silver resistance (*SilABCDPRS*), and tellurium resistance (*terABCDZ*), plasmid pCR2021_IncFIB also harbored the virulence-related genes *iroBCDN* and *iucABCD-iutA* (encoding aerobactin and transporter). Finally, the carbapenemase gene *bla*_NDM-5_, responsible for carbapenem resistance in this strain, was found on the pCR2021_IncX3 plasmid. Furthermore, the plasmid harbored *VirB1-11* (excluding *VirB3* and *VirB7*) and *VirD4*, indicating that the plasmid carries a relatively intact *VirB/D4* system, which is characteristic of the type IV secretion system (T4SS) (Table 2).

### Genetic environment analysis of plasmid drug-resistance genes

**Fig 2:**
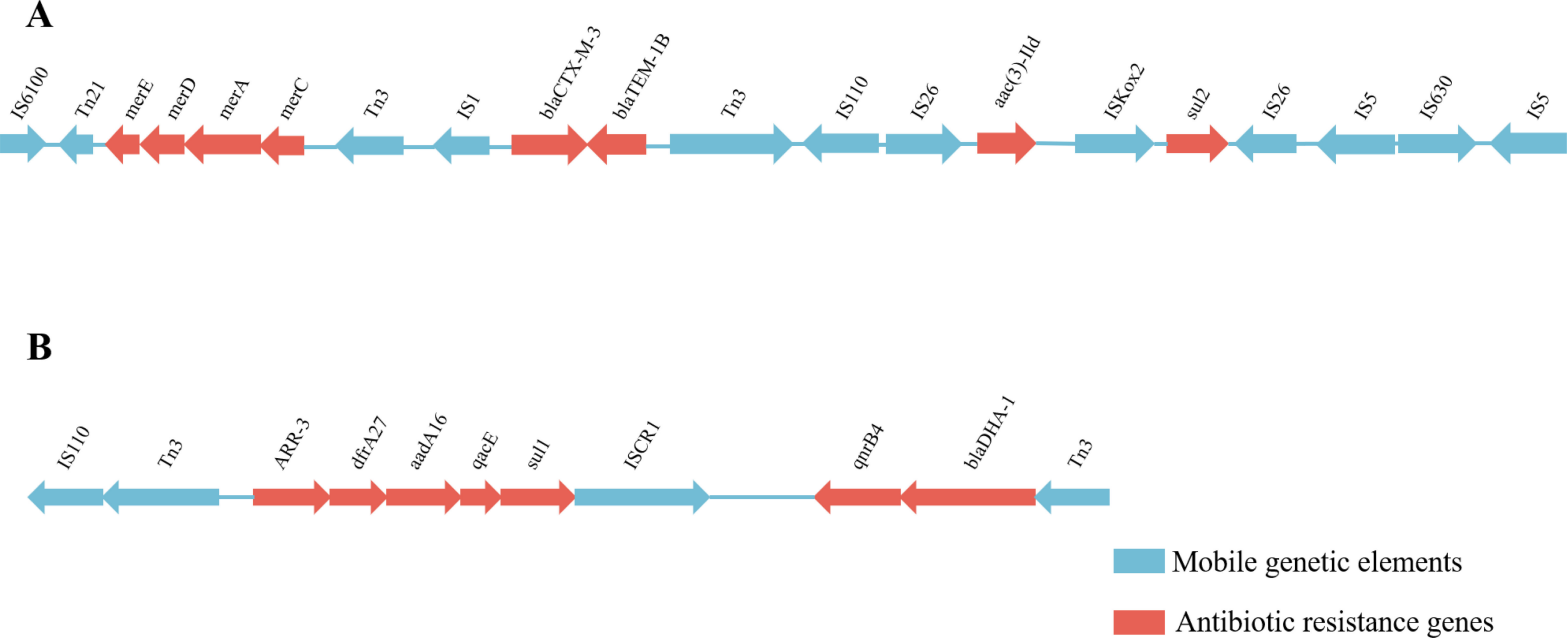
Genetic structure of the pCR2021_IncFII drug-resistance gene. (**A**) Structural annotation of a 41.8 kb region containing the resistance genes *merACDE*, *bla*_CTM-M-3_, *bla*_TEM-1B_, *aac (3)-lld*, and *sul2*. (**B**) Structural annotation of a 32.3 kb region containing the drug-resistance genes *ARR-3*, *dfrA27*, *aadA16*, *qacE*, *sul1*, *qnrB4*, and *bla*_DHA-1_.

Based on the results of whole gene sequencing and drug-resistance gene prediction, all three plasmids of strain CR2021 were found to harbor drug-resistance genes, predominantly located on plasmids pCR2021_IncFII and pCR2021_IncX3. Plasmid pCR2021_IncFIB only regulated the strain’s resistance to copper, silver, and tellurium. Plasmid pCR2021_IncFII harbored multiple resistance genes organized into two segments with multiple mobile elements, sized at 41.8 kb and 32.3 kb, respectively. The *merACDE*, *bla*_CTX-M-3_, *bla*_TEM-1B_, *aac (3)-Ild*, and *sul2* genes were located in the 41.8 kb fragment. The *bla*_CTX-M-3_ and *bla*_TEM-1B_ genes were located near the central part of this segment, and their upstream and downstream regions were connected to the mobile elements Tn*3*-IS*1* and Tn*3*-IS*110*-IS*26*, respectively. The 5′ end of the Tn*3* transposon was fused with IS*6100*-Tn*21-merACDE*, while the 3′ end IS*26* was directly connected to *aac (3)-lld*-IS*kox2-sul2*-IS*26*-IS*5*-IS*630*-IS*5* (Fig. 2A). The genes for rifampicin resistance (*ARR-3*), trimethoprim resistance (*dfrA27*), aminoglycoside resistance (*aadA16*), quaternary amine resistance (*qacE*), sulfanilamide resistance (*sul1*), quinolone resistance (*qnrB4*), and *β*-lactam resistance (*bla*_DHA-1_) were located on a 32.3 kb-size fragment. The *ARR-3*, *dfrA27*, *aadA16*, *qacE*, and *sul1* directly connected the main body. The 5′ end and the IS*110*-Tn*3* element were linked together, and the 3′ end was connected to the *qnrB4-bla*_DHA-1_-Tn*3* gene combination via IS*CR1* (Fig. 2B).

**Fig 3:**
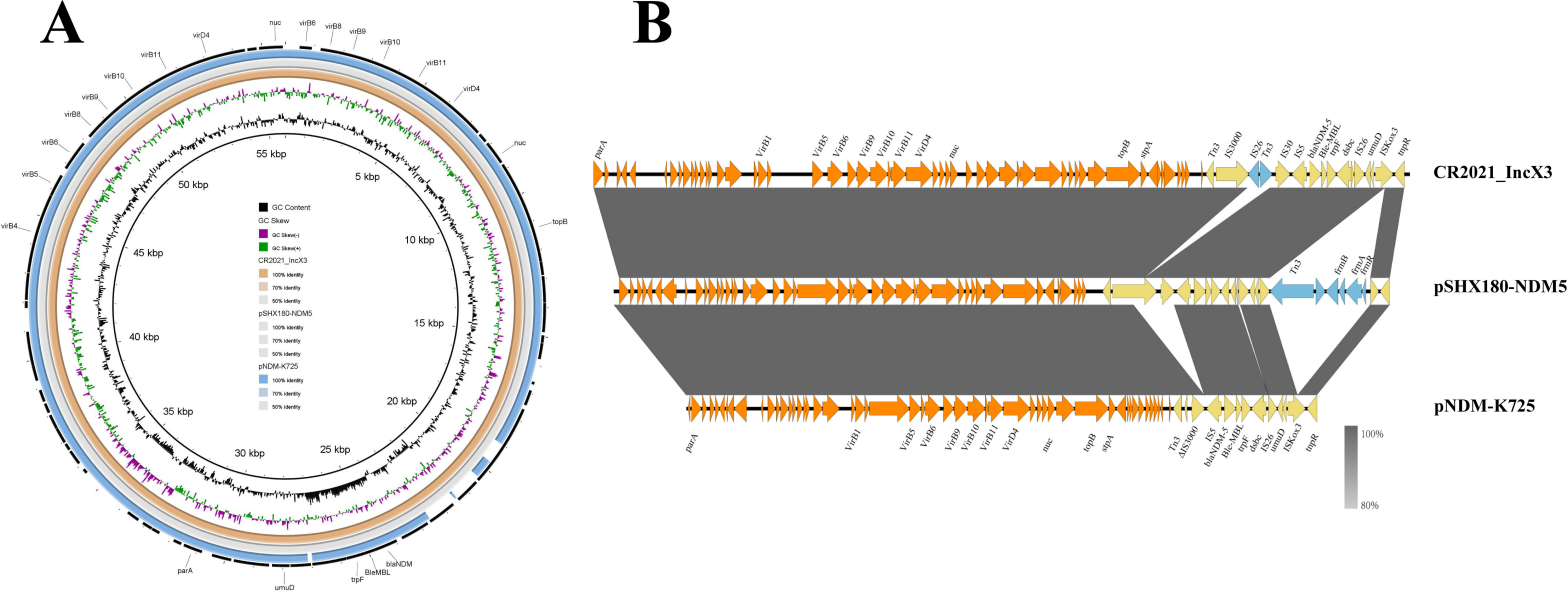
Comparative circular and linear analysis of plasmids pCR2021_IncX3, pSHX180-NDM5, and pNDM-K725 revealed 99% identity as well as 87% and 100% coverage, respectively. (**A**) Circular structure diagram comparing plasmid pCR2021_IncX3 and two other IncX3 plasmids (pSHX180-NDM5 and pNDM-K725) originating from *K. pneumoniae*. Plasmid pCR2021_IncX3 served as the reference strain. The outermost ring was the primary gene carried by plasmid pCR2021_IncX3. Plasmid pNDM-K725 is represented by a blue circle, while plasmids pSHX180-NDM5 and pCR2021_IncX3 are represented by gray and orange circles, respectively. (**B**) Linear comparative analysis of the genetic environment among plasmids pCR2021_IncX3, pSHX180-NDM5, and pNDM-K725 *bla*_NDM-5_. Arrows represent CDS, with yellow arrows representing mobile elements near the *bla*_NDM-5_ genes, blue arrows representing differential genes, and shaded regions representing the extent of plasmid similarity.

Plasmid pCR2021_IncX3 carried a single resistance gene, *bla*_NDM-5_, often located on plasmid IncX3, facilitating its widespread dissemination among *K. pneumoniae*. Two IncX3 plasmids were selected for comparative genetic characterization. Plasmid BLAST comparison analysis showed that the plasmid pCR2021_IncX3 exhibited 99% identity with pSHX180-NDM5 and pNDM-K725, with coverages of 87% and 100%, respectively, when used as the reference plasmid. Only a few genetic differences were found upstream of *bla*_NDM-5_ on the plasmid pNDM-K725 (Fig. 3A). To more comprehensively assess similarities and differences among the three plasmids, we conducted a linear genomic comparison analysis. The results showed that *bla*_NDM-5_ was located on the Tn*3* transposon module, flanked by numerous mobile elements on both sides. It constituted the Tn*3*-IS*3000*-IS*26*-Tn*3*-IS*30*-IS*5-bla*_NDM-5_-*Ble-MBL-trpF-dsbc*-IS*26-umuD*-IS*Kox3* gene combination. Differential gene analysis revealed that, compared to pSHX180-NDM5, the pNDM-K725 5′ end IS*3000* was truncated (ΔIS*3000*) and lacked IS*30*, while pCR2021_IncX3 exhibited an additional IS*26*-Tn*3* module compared to pSHX180-NDM5 IS*3000*-IS*30*. Besides differences in insertion elements, pSHX180-NDM5 harbored a region encoding the *frmRAB* operon, facilitating bacterial formaldehyde sensing and detoxification effects, features absent in the plasmids pCR2021_IncX3 and pNDM-K725. In addition, all three plasmids carried a relatively intact T4SS (Fig. 3B), which mediates bacterial conjugation and subsequently promotes horizontal gene transfer.

### Analysis and verification of bacterial virulence characteristics

**Fig 4:**
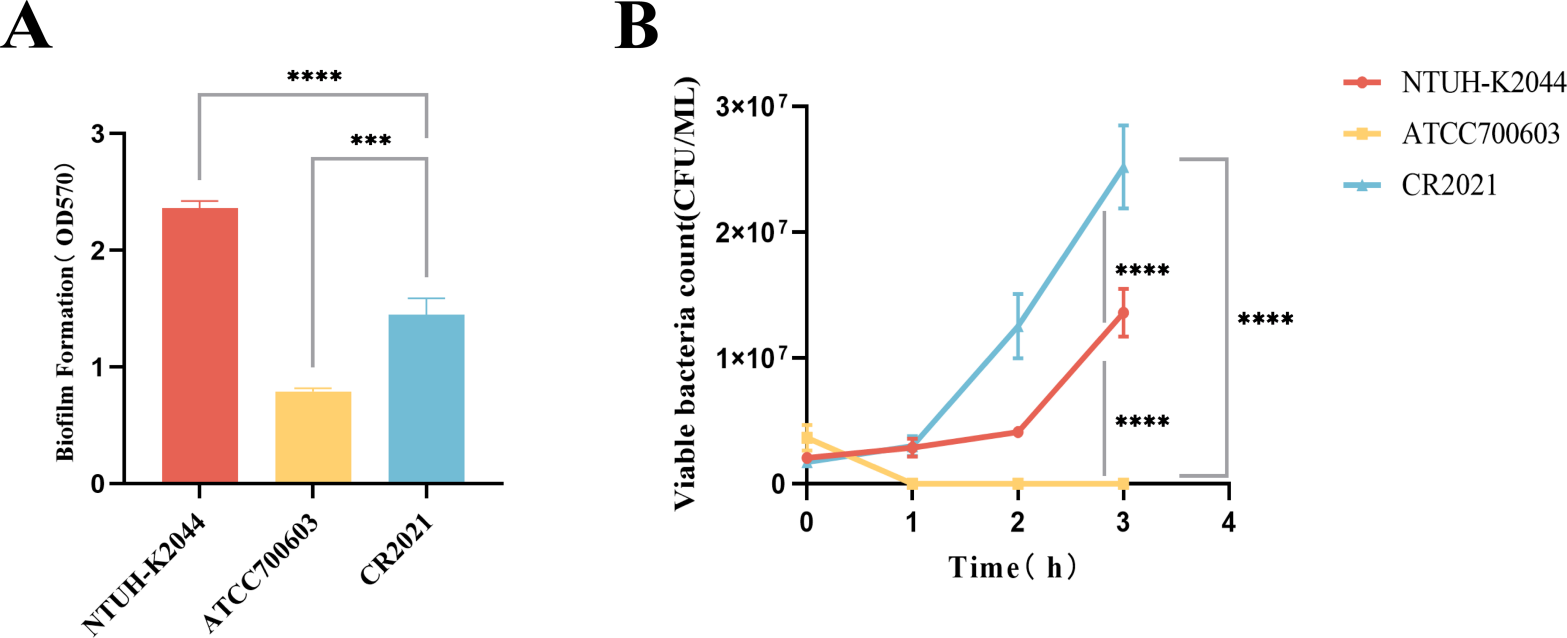
Strain CR2021 biofilm formation and serum killing tests. (**A**) Bacterial biofilm formation test. The amount of biofilm formed was determined by measuring the absorbance at 570 nm using an enzyme-labeled instrument. Strain CR2021 exhibited a significantly different amount of biofilm formation compared to the positive control NTUH-K2044 and the negative control ATCC700603. (**B**) Serum killing test. Bacterial cultures were incubated with the serum from healthy individuals for 3 h to assess the tolerance of the strain to the serum. NTUH-K2044 served as the serum-tolerance control, and ATCC700603 served as the serum-sensitive control. ***P < 0.001, ****P < 0.0001.

**Fig 5:**
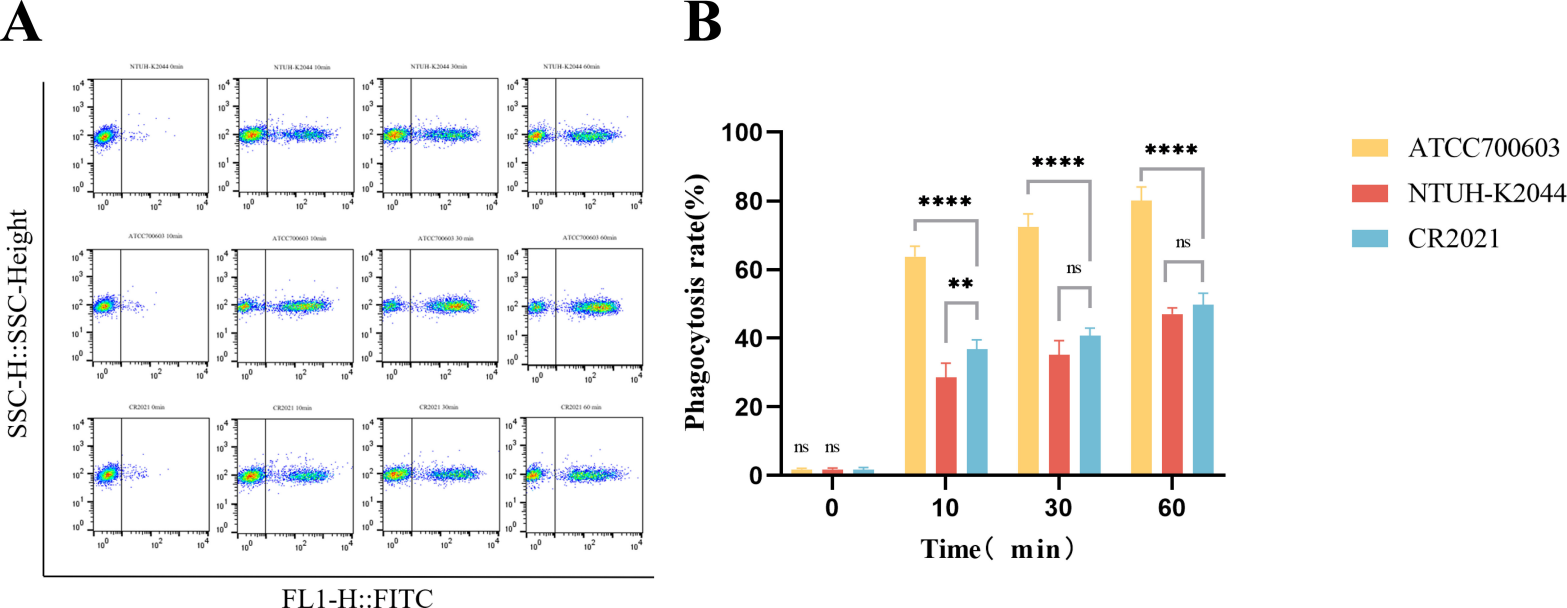
Strain CR2021 anti-human neutrophil phagocytosis test. (**A**) Flow cytometry scatter plot depicting fluorescein isothiocyanate (FITC) fluorescence intensity on the horizontal axis and the lateral scattering light parameter (SSC) on the vertical axis. (**B**) Histogram showing results of the anti-human neutrophil phagocytosis test. NTUH-K2044 served as the positive control for anti-phagocytosis, while ATCC700603 served as the negative control for anti-phagocytosis. **P < 0.01, ****P < 0.0001.

**Fig 6:**
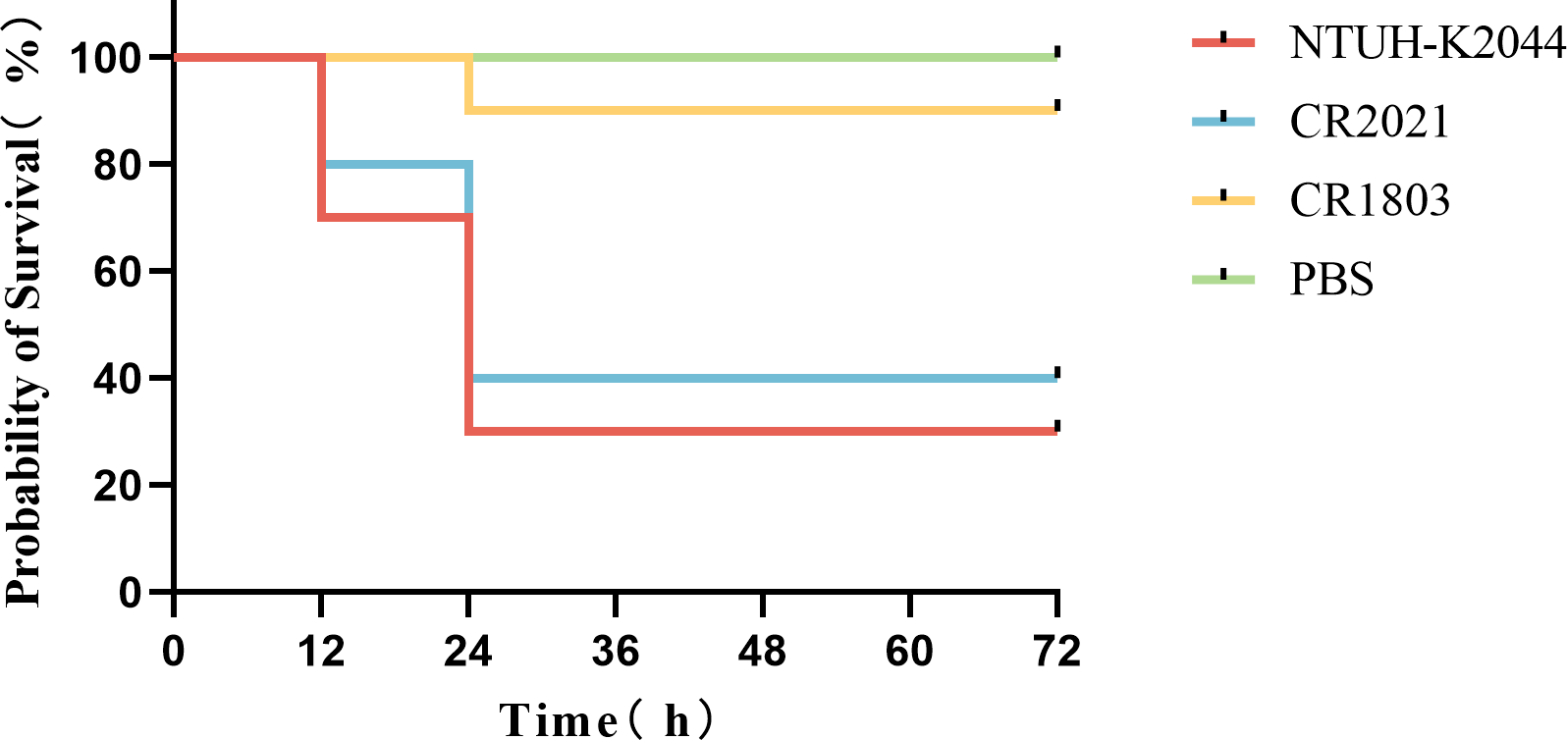
Larvae infection model of *Galleria mellonella* was used to verify the virulence of strain CR2021 *in vivo*, with NTUH-K2044 serving as a highly toxic control, CR1803 as a low-toxicity control, and sterile Phosphate Belanced Solution (PBS) as a blank control. The larvae were injected with bacteria (1 × 10^7^ CFU/mL), monitored for 72 h, and survival curves were plotted. Strain CR2021 exhibited comparable virulence to NTUH-K2044 in the *G. mellonella* larvae infection model *G. mellonella*.

The prediction of virulence-related genes showed that strain CR2021’s chromosome harbored six virulence genes, including the allantoin gene (*allS*), *fimABCDEFGHI* (encoding type I fimbrial), *mrkABCDFHIJ* (encoding type III fimbrial), yersiniabactin (*ybt*, *fyu*), salmonellin (*iroBD*), and enterobactin (*entABCDEF*). Additionally, numerous genes encoding capsular and lipopolysaccharide components closely associated with anti-serum killing and anti-phagocytosis effects were observed. Moreover, the plasmid pCR2021_IncFIB harbored the virulence-related genes *iroBCDN* and *iucABCD-iutA*. Therefore, we performed the biofilm formation test, serum killing test, anti-human neutrophil phagocytosis test, and larvae infection model of *Galleria mellonella* to further analyze the virulence characteristics of this strain. The results of the biofilm formation test showed that strain CR2021 exhibited significant differences in biofilm formation compared to the positive control NTUH-K2044 and the negative control ATCC700603 (*P* < 0.001) (Fig. 4A). In the serum killing test, the bacterial concentration of strain CR2021 gradually increased within 3 h of incubation with serum from healthy individuals, demonstrating obvious serum tolerance (Fig. 4B). During the anti-human neutrophil phagocytosis test, following incubation with healthy neutrophils for 1 h, the phagocytosis rate of neutrophils toward CR2021 was approximately 50%, significantly lower than that observed with ATCC700603 in the absence of anti-neutrophilic phagocytosis (*P* < 0.0001) (Fig. 5A and B). Finally, the *in vivo* virulence of strain CR2021 was verified using a *G. mellonella* larvae infection model. The results showed that upon injecting a bacterial solution (1 × 107 CFU/mL) into *G. mellonella* larvae, the larval survival rate was 40% at 72 h, which was comparable to the 30% survival rate observed with NTUH-K2044, a highly toxic strain which was used as the control. Both rates were significantly lower than the 90% survival rate observed with the low-toxicity control, CR1803 (Fig. 6).

## DISCUSSION

The widespread dissemination of plasmid-mediated drug-resistance genes has facilitated the emergence of CR-hvKP, which often exhibits multidrug resistance. The emergence of CR-hvKP complicates treatment significantly (10). In this study, a novel sequence type of CR-hvKP, CR2021, was characterized. Drug resistance and virulence characteristics were determined through whole-genome sequencing. CR2021 belongs to a novel sequence type, ST6417, which was identified for the first time, and its capsule serotype was determined to be the typical hypervirulent serotype, K1. ST6417 is a single locus variant of ST23, exhibiting a C-to-A mutation at locus 239 of *tonB*. ST23 is the predominant sequence type among hvKP in China, and most hvKP strains of ST23 exhibit susceptibility to drugs (11). However, CR2021, isolated from a blood sample of a 78-year-old male patient with bacteremia, exhibited resistance to all first-line antibiotics except for tigecycline and amikacin. This strain causes bloodstream infections that result in septic shock, eventually leading to death. In China, the widely prevalent MDR *K. pneumoniae* often manifests as ST11 and its variants. The evolution of ST11 from CR-non-hvKP to CR-hvKP through the acquisition of virulence plasmids is common, whereas the evolution of ST23 to CR-hvKP is rare (12). Moreover, in recent years, the detection rate of hvKP has been increasing, potentially replacing cKP as the predominant pathogen (1). Therefore, conducting in-depth research on ST6417 is necessary to determine the molecular mechanisms underlying the transformation of hvKP to CR-hvKP.

To adapt to their living environment, bacterial genomes undergo continuous changes, especially under antibiotic selection pressure, which prompts adjustments in gene expression through various mechanisms to confer resistance to antibiotics. It is worth noting that such genomic changes can propagate to other bacteria through the transfer of mobile drug-resistance elements, thereby resulting in the emergence of more drug-resistant bacteria (13). The strain CR2021 examined in this study comprises a single chromosome and three plasmids. Gene prediction analysis indicated that the plasmid pCR2021_IncFII likely plays the predominant role in the strain’s multidrug resistance, as it harbors up to eight drug-resistance genes. Comparison with data from the PLSDB database revealed that the plasmid exhibited 99% sequence similarity with plasmid pKP18-31-IMP, which also originates from *K. pneumoniae*. A 32.3 kb drug-resistant region in plasmid pCR2021_IncFII showed high similarity to the region near *bla*_IMP-4_ in pKP18-31-IMP. pKP18-31-IMP carries two carbapenemase-coding genes, *bla*_KPC-2_ and *bla*_IMP-4_, while plasmid pCR2021_IncFII lacks carbapenemase-coding genes but harbors two *β*-lactamase genes, *bla*_CTX-M-3_ and *bla*_TEM-1B_. Previous studies have reported cases where acquisition and loss of carbapenemase-coding genes occurred simultaneously during treatment; another study indicated that deletion of carbapenemase-coding genes results in reduced drug resistance in strains, indicating that such deletions are not rare. Further investigation into whether the proximity of mobile elements to resistance genes contributes to these phenomena is warranted (14, 15).

In this study, despite the absence of carbapenemase-coding genes in plasmid pCR2021_IncFII, CR2021 retained a carbapenem resistance phenotype because of the presence of *bla*_NDM-5_ on plasmid pCR2021_IncX3. Among carbapenemase-coding genes, *bla*_KPC_ is considered the dominant gene (16). However, *bla*_NDM_ demonstrates a robust hydrolytic ability. Since its initial report, *bla*_NDM_ has been disseminated worldwide, and diverse *bla*_NDM_ drug-resistance gene variants have been detected. Following the discovery of *bla*_NDM-5_ in MDR *Escherichia coli* in England, China, the United States, Spain, and other countries, reports on *bla*_NDM-5_ have also been published, indicating that the *bla*_NDM-5_ resistance gene exhibits outstanding transferability (17–20). Previous studies have demonstrated that *bla*_NDM-5_ is often harbored by IncX3 plasmids and exhibits high stability (21). Consistent with previous findings, the *bla*_NDM-5_ drug-resistance gene in this study was also located on the IncX3 plasmid, located within a highly mobile region. In another related study focusing on *bla*_NDM-5_, this drug-resistance gene was found on the plasmid pSHX180-NDM5. The strain carrying this plasmid belonged to a novel sequence type, ST4523, arising from mutations in the ST11 housekeeping genes *mdh* and *phoE* (22). Comparison between plasmid pSHX180-NDM5 and plasmid pCR2021_IncX3 showed that the genetic environment of *bla*_NDM-5_ of the two strains was highly similar. However, plasmid pCR2021_IncX3 contains an additional IS*26*-Tn*3* gene combination, suggesting that *bla*_NDM-5_ in this plasmid may be more susceptible to transfer. Another study focusing on the IncX3 plasmid carrying the *bla*_NDM-5_ resistance gene showed widespread prevalence among enterobacteria causing childhood diseases. These findings indicate that carbapenem-resistant *K. pneumoniae* (CR-KP) mediated by such plasmids differs from traditional *K. pneumoniae*, i.e., there was no age difference in the likelihood of contracting them (23, 24). The aforementioned plasmid, pNDM-K725, was selected and compared with pCR2021_IncX3 in this study. The results showed that their backbone regions were highly similar. However, compared with pCR2021_IncX3, pNDM-K725 exhibited a truncated IS*3000* and lacked the IS*26*-Tn*3*-IS*30* flank region. Additionally, T4SS, which is closely associated with plasmid-coupled transfer, was found in all three plasmids (pCR2021_IncX3, pSHX180-NDM5, and pNDM-K725), suggesting a robust self-transfer capability that increases the possibility of drug-resistance gene transmission (25). However, in contrast to plasmid pSHX180-NDM5, plasmids pCR2021_IncX3, and pNDM-K725 did not contain the region encoding the *frmRAB* operon, indicating that strains carrying the latter two plasmids may lack the ability to sense formaldehyde.

The virulence of *K. pneumoniae* is closely associated with genes encoding capsular polysaccharides, lipopolysaccharides, fimbriae, and iron uptake systems (26). In this study, genes encoding type I fimbrial, type III fimbrial, enterobactin, salmonellin, and yersiniabactin, and numerous genes encoding capsular polysaccharide and lipopolysaccharide, were found on the chromosome of CR2021. Moreover, genes encoding salmonellin and aerobactin were identified on the pCR2021_IncFIB plasmid. Therefore, we hypothesized that virulence genes located on the chromosome and plasmid pCR2021_IncFIB co-mediate the high virulence phenotype of strain CR2021. Subsequent biofilm formation, serum killing, and anti-human neutrophil phagocytosis tests confirmed this conjecture, with further validation in a *G. mellonella* larvae infection model. The multi-organ infection induced by this hypervirulent strain contributed to the development of multiple organ failure, ultimately leading to the patient’s demise.

In conclusion, CR2021, a novel ST6417 K1 serotype strain, closely related to the ST23 K1 serotype hvKP, was characterized in this study. While CR-hvKP arising from ST11 CR-KP and its variants currently dominate in China and have been extensively studied, CR-hvKP arising from hvKP has received relatively less attention. The emergence of this strain suggests that more extensive and in-depth studies are needed to develop appropriate measures for controlling the prevalence of CR-hvKP.

## MATERIALS AND METHODS

### Bacterial strains

CR2021 was isolated from a blood sample obtained from a 78-year-old male patient. The blood sample was inoculated onto sterile Petri dishes following a positive bacterial culture alert. The isolate was identified via matrix-assisted laser desorption ionization-time of flight mass spectrometry.

### *In vitro* drug-sensitivity test

The MICs of tigecycline, cefuroxime sodium piperacillin/tazobactam, cefepime, cefuroxime axetil, cefoxitin, cefoperazone/sulbactam, ceftazidime, ceftriaxone, ertapenem, imipenem, amikacin, sulfamethoxazole, levofloxacin, and moxicillin/clavulanic acid were determined using Vitek2 with a GN334 card. The Kirby–Bauer disc diffusion method was used to assess bacterial susceptibility against gentamicin, aztreonam, ampicillin/sulbactam, and meropenem. *E. coli* ATCC25922 and *K. pneumoniae* ATCC700603 served as quality control strains. Antimicrobial susceptibility was determined using breakpoints recommended in the Clinical and Laboratory Standards Institute (CLSI) guidelines (CLSI M100 2023). The tigecycline MIC was interpreted according to the breakpoint specified by the FDA (27).

### MLST and capsular serotyping

MLST and capsule serotype were amplified through PCR, amplifying seven housekeeping genes and the *Wzi* gene (https://bigsdb.pasteur.fr/). Gene sequences were acquired via first-generation Sanger sequencing and subsequently uploaded to the Pasteur Institute’s website in France for the final determination of MLST type and capsule serotype.

### Whole-genome sequencing and bioinformatic analysis

The entire bacterial genome was sequenced using second-generation Illumina and third-generation PacBio technologies. The assembly software unicycler v0.4.8 was used for third-generation sequence assembly. Assembly results for the chromosomal genome were predicted using Prodigal, while GeneMarkS was used for the plasmid genome. PlasFlow software was used to identify plasmids. BLAST and the PLSDB database were used for plasmid annotation. Resfinder 4.1.0 was employed to predict drug-resistance genes in the genome, and virulence genes were identified in the virulence factor database VFDB using Diamond software. ISEScan software was used to identify insertion sequence elements within the genome. A circular genome map was generated using Circos software, and a plasmid comparison circular map was generated using BRIG software. Genome linear comparison analysis and mapping were performed using EasyFigure 2.2.5.

### Biofilm formation test

The biofilm formation ability of strain CR2021 was investigated using a biofilm formation test, with minor modifications as described above (28). A mixture containing 190 µL of LB broth and 10 µL of bacterial solution (1 × 10^6^ CFU/mL) was added to each well of a 96-well polystyrene plate. Six replicate wells were prepared and incubated at a constant temperature of 37°C for 18 h. Subsequently, the bacterial solution was removed, and each well was washed three times with 200 µL of distilled water. Then, 225 µL of 0.1% crystal violet dye was added, allowed to stand for 20 min, and the wells were washed three times. Subsequently, 225 µL of anhydrous ethanol was added for decolorization for 20 min. Next, the absorbance of 125 uL of the decolorized products at 570 nm was determined using an enzyme labeling instrument. NTUH-K2044 served as the positive control, and ATCC700603 served as the negative control, respectively. The experiment was repeated at least three times.

### Serum killing test

The serum killing test was performed as described previously, with minor modifications (29). A single colony was cultured in LB broth and incubated at a constant temperature shaker at 37°C overnight. An aliquot (200 µL) of the overnight culture was diluted 1:100 and cultured to the mid-logarithmic phase, achieving a concentration of 1 × 10^7^ CFU/mL. A 25 µL aliquot of bacterial solution was mixed with 75 µL of serum from a healthy individual and incubated at 37°C for 3 h. Following proper dilution of 10 µL of culture at 0 min, 1 h, 2 h, and 3 h, the culture was densely coated on Columbia blood agar plates, and the plates were incubated in a constant temperature incubator at 37°C for 24 h. NTUH-K2044 and ATCC700603 were used as positive and negative controls, respectively. The experiment was repeated at least three times.

### Anti-human neutrophil phagocytosis test

To explore whether strain CR2021 could evade neutrophil phagocytosis, we conducted an anti-human neutrophil phagocytosis test, as described above (30). A bacterial solution of a suitable concentration was incubated in a water bath at 70°C for 1 h. Subsequently, fluorescein isothiocyanate (FITC) was added at a final concentration of 0.1 mg/mL and incubated at 25°C for 1 h. Neutrophils were isolated from healthy individuals using PolymorphPrep, following the manufacturer’s instructions. After isolation, the Trypan Blue staining solution was used to assess viability and count cells. Finally, FITC-labeled bacteria and healthy human neutrophils were prepared at a multiplicity of infection of 40:1 and incubated on a constant-temperature shaking table at 37°C for 1 h. Samples were retrieved and washed at 0, 10, 30, and 60 min, and the proportion of FITC-positive neutrophils, indicative of phagocytosis, was determined through flow cytometry. In this test, NTUH-K2044 served as the positive control for anti-phagocytosis, while ATCC700603 served as the negative control for anti-phagocytosis. The experiment was repeated at least three times.

### Larvae infection model of *G. mellonella*

The *in vivo* pathogenicity of CR2021 was determined using the experimental method outlined above (31). After resuscitating strain CR2021, a bacterial solution was prepared with a concentration of 0.5 MCF, which was diluted 10-fold. Subsequently, 10 µL of this diluted solution was injected into the larvae. The injected larvae were then incubated in a constant temperature incubator at 37°C, with observations recorded every 12 h regarding larval survival. The experiment extended over 72 h, with 10 larvae in each group. NTUH-K2044 served as the high-toxicity control in this experiment, while ST11-K47 CR-KP CR1803 collected from our laboratory served as the low-toxicity control. Sterile PBS was used as the blank control.

## Data Availability

The raw sequencing data of strain CR2021 were deposited in the GenBank database (https://www.ncbi.nlm.nih.gov/genbank/) under the accession numbers CP147868–CP147871. The other plasmids used in this study included pSHX180-NDM5:CP094514.1, pNDM-K725:MK450348, and pKP18-31-IMP:MN661402.1.

## SUPPLEMENTAL MATERIAL

Figure S1 spectrum.00984-24-s0001.docx 12h growth curve of bacteria.

ASM does not own the copyrights to Supplemental Material that may be linked to, or accessed through, an article. The authors have granted ASM a non-exclusive, world-wide license to publish the Supplemental Material files. Please contact the corresponding author directly for reuse.
