# Supplementary material for: Emergence of a novel sequence type carbapenem-resistant hypervirulent Klebsiella pneumoniae ST6417 harboring blaNDM-5 on the lncX3 plasmid
Source: Microbiol Spectr. 2024 Aug 20;12(10):e00984-24. doi: 10.1128/spectrum.00984-24 (PMC11448260; doi:10.1128/spectrum.00984-24)
Supplement: Figure S1 — 12h growth curve of bacteria. [file spectrum.00984-24-s0001.docx]

**Method:** A single colony was cultured in LB broth and incubated at a constant temperature shaker at 37 °C overnight. An aliquot (200 µL) of the overnight culture was diluted 1:100 and cultured to mid-logarithmic phase, add 100ul bacterial solution to 10ml LB broth medium, immediately take 125ul mixture to measure its absorbance value at 570nm, and continuously measure for 12h.

**Result:**


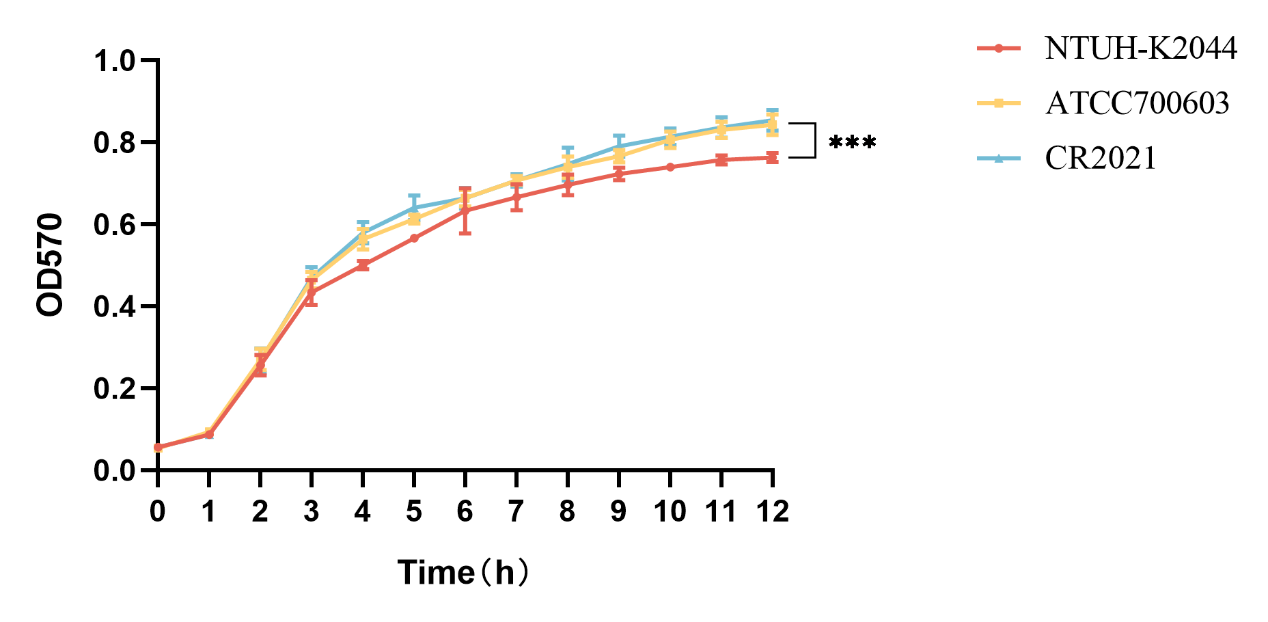


In the 12h growth curve of bacteria, NTUH-K2044 was ST23 hvKP, ATCC700603 was low virulence but ESBL positive strain, and CR2021 was ST6417 CR-hvKP carrying *bla*_NDM-5_ resistance gene.
